# Inhibition of HIV early replication by the p53 and its downstream gene p21

**DOI:** 10.1186/s12985-018-0959-x

**Published:** 2018-03-27

**Authors:** Binshan Shi, Hamayun J. Sharifi, Sara DiGrigoli, Michaela Kinnetz, Katie Mellon, Wenwei Hu, Carlos M. C. de Noronha

**Affiliations:** 1 Department of Basic and Clinical Sciences, Albany College of Pharmacy and Health Sciences, 106 New Scotland Ave, Albany, NY 12208 USA; 2 Rutgers Cancer Institute of New Jersey, Rutgers the State University of New Jersey, New Brunswick, NJ 08903 USA; 3 Center for Immunology and Microbial Disease, Albany Medical College, Albany, New York, Albany, NY 12208 USA

**Keywords:** p53, p21, HIV-1, HIV-2, Reverse transcription, Cell cycle, SAMHD1, RNR2

## Abstract

**Background:**

The tumor suppressor gene p53 has been found to suppress HIV infection by various mechanisms, but the inhibition of HIV at an early stage of replication by host cell p53 and its downstream gene p21 has not been well studied.

**Method:**

VSV-G pseudotyped HIV-1 or HIV-2 viruses with GFP or luciferase reporter gene were used to infect HCT116 p53^+/+^ cells, HCT116 p53^−/−^ cells and hMDMs. The infections were detected by flow cytometry or measured by luciferase assay. Reverse transcription products were quantified by a TaqMan real time PCR. siRNA knockdown experiments were applied to study potential roles of p53 and p21 genes in their restriction to HIV infection. Western blot experiments were used to analyze changes in gene expression.

**Results:**

The infection of HIV-1 was inhibited in HCT116 p53^+/+^ cells in comparison to HCT116 p53^−/−^ cells. The fold of inhibition was largely increased when cell cycle switched from cycling to non-cycling status. Further analysis showed that both p53 and p21 expressions were upregulated in non-cycling HCT116 p53^+/+^ cells and HIV-1 reverse transcription was subsequently inhibited. siRNA knockdown of either p53 or p21 rescued HIV-1 reverse transcription from the inhibition in non-cycling HCT116 p53^+/+^ cells. It was identified that the observed restrictions by p53 and p21 were associated with the suppression of RNR2 expression and phosphorylation of SAMHD1. These observations were confirmed by using siRNA knockdown experiments. In addition, p53 also inhibited HIV-2 infection in HCT116 p53^+/+^ cells and siRNA knockdown of p21 increased HIV-2 infection in hMDMs. Finally the expressions of p53 and p21 were found to be induced in hMDMs shortly after HIV-1 infection.

**Conclusions:**

The p53 and its downstream gene p21 interfere with HIV early stage of replication in non-cycling cells and hMDMs.

## Background

The tumor suppressor gene p53 has been suggested to play an important role in the restriction of the human immunodeficiency virus type 1 (HIV-1) infection for decades. p53 is activated in human immune cells after HIV-1 infection [1–3], and p53 expression is induced by type I interferons (IFN-α/β) after viral infection [2, 4]. A variety of mechanisms have been proposed to reveal p53 mediated restrictions to HIV infection. Early studies found that p53 inhibited HIV-1 long terminal repeat (LTR) promoter activity and repressed transcription from the HIV-1 proviral genome [5–7]. p53 was also found to suppress Tat, a major transactivator of HIV-1 [8]. More recently Yoon et al. reported that p53 induced the expression of PKR, and then PKR inactivated HIV-1 Tat by phosphorylation. Many other works reported that HIV-1 infection caused immune cell’s death by inducing p53 dependent apoptosis [1, 9–12]. Additionally, it was postulated that p53 might impact HIV-1 reverse transcriptase function [13, 14], but detailed mechanism remains to be determined at the cellular level. The evidence that p53 inhibits HIV-1 at an early stage of replication has not been reported previously.

One of the p53 downstream genes, the cyclin-dependent kinase inhibitor p21^Waf1/Cip1^ (referred to hereafter as p21) has been documented for its role in antiretroviral infection [15–21]. The expression of p21 in human macrophages was induced after HIV-1 infection [19]. Upregulation of p21 was also found in CD4^+^ T cells from elite controllers, a unique group of HIV-1–infected individuals with undetectable HIV-1 replication in the absence of antiretroviral therapy [18, 22]. siRNA knockdown of p21 resulted in increased HIV-1 infection [22]. Both Allouch et al and Pauls et al showed that p21 inhibited HIV-1 reverse transcription in macrophages through regulating level of cellular dNTPs [17, 20]. Other data indicated that inhibition of HIV-1 reverse transcription by p21 might not be directly related with regulating the level of cellular dNTPs. Leng et al. showed that p21 inhibited CDK2-dependent phosphorylation of HIV-1 reverse transcriptase, which reduced the efficacy of HIV-1 reverse transcription [18]. Zhang et al showed that p21 prevented viral DNA integration [23]. Others reported that the restriction to HIV-1 infection by p21 was associated with viral protein Vpr [24, 25]. It was also found that p21 inhibited HIV-2 and SIV infection [16].

It was found previously by our group that p53 inhibited reverse transcription of MLV vector based retrovirus in non-cycling cells through its downstream gene p21 [26]. It was investigated in this study whether the p53 dependent host restriction to retrovirus also applies to HIV infection by using human colorectal cancer HCT116 p53^+/+^ and HCT116 p53^−/−^ cell lines and primary human monocyte derived macrophages (hMDMs). Interestingly, p53 and its downstream gene p21 were found upregulated in hMDMs shortly after HIV-1 infection. Our results strongly suggest their antiretroviral roles at an early stage of HIV replication.

## Methods

### Cell culture and cell viability

Human colorectal cancer HCT116 p53^+/+^ and HCT116 p53^−/−^ cell lines were generous gifts from Dr. B. Vogelstein. HEK-293 cells and TZM-bl cells were obtained from NIH AIDS Reagent Program (Germantown, MD, USA). HCT116 p53^+/+^, HCT116 p53^−/−^, HEK-293 and TZM-bl were propagated in Dulbecco’s modified Eagle’s medium (DMEM) (Life Technologies, Grand Island, NY, USA) supplemented with 10% fetal bovine serum (FBS), 2 mM L-Glutamine, 100 units/ml of penicillin and 100 μg/ml of streptomycin at 37 °C with 5% CO_2_. A Countess II Automated Cell Counter (Thermos Fisher Scientific, Waltham, MA, USA) was used to count the number of cells in experiments as designed. Elutriated human monocytes were obtained from the University of Nebraska Medical Center. Donors were de-identified with consent procedures based on the anonymity. IRB approval has been obtained from the Committee on Research at Albany College of Pharmacy and Health Science. Monocytes were differentiated into macrophages (hMDMs) for 10–14 days in DMEM supplemented with 10% human AB serum (VWR, Radnor, PA, USA) by following procedures described previously [27]. Non-cycling HCT116 p53^+/+^ and HCT116 p53^−/−^ cells were prepared by 24 h serum starvation. Both cycling and non-cycling cells were tested for cell viability by using trypan blue exclusion assay, and cell death was also measured by WST-1 assay (Roche, Indianapolis, IN, USA). The percentage of live cells was counted by the Countess II Automated Cell Counter (Thermos Fisher Scientific, Waltham, MA, USA) after cells were stained by the Trypan Blue solution (Thermos Fisher Scientific, Waltham, MA, USA). WST-1 assay (Roche) was performed by following kit instruction and O.D. was measured by Eppendorf Plate Reader AF2200 (Eppendorf, Hamburg Germany).

### Virus preparation and cell infection

HIV VSV-G-pseudotyped viruses were produced by transient cotransfection of HEK293 cells with proviral HIV-1 or HIV-2 plasmids together with a vesicular stomatitis virus G protein (VSV-G) expression vector pVSV-G (Clontech Laboratories, Inc., Mountain View, CA, USA) by using X-tremeGENE 9 DNA Transfection Reagent (Roche, Indianapolis, IN, USA). HIV-1 plasmids pNL4–3 *env(−)nef(−)gfp(+)* was a gift from Dr. Vicente Planelles, pNL4–3 *env(−)nef(−)luc(+)* was a gift from Dr. Nathaniel Landau and HIV-2 *luc(+)* was a gift from Dr. Lee Ratner. Supernatants containing pseudotyped viruses were harvested 48 h after transfection, passed through 0.45-nm-pore-size filters, and stored at − 80 °C. Viral titers were determined by serial dilution on the TZM-bl indicator cell line as previously described [28]. 1 × 10^5^ cells/well were seeded in a 24 well plate for infection of HCT116 p53^+/+^ and HCT116 p53^−/−^ cells. For non-cycling cells, the complete medium was replaced with DMEM medium without FBS after 24 h, and cells were infected after another 24 h. For cycling cells the medium was replaced with fresh complete medium after 24 h. At time of the infection, cell numbers of paired HCT116 p53^+/+^ and HCT116 p53^−/−^ cells were counted by a Countess II Automated Cell Counter (Thermos Fisher Scientific, Waltham, MA, USA), the same MOI was used for infection in both cells. 0.5 × 10^6^ hMDMs cultured in 24 well plates were used for HIV infection and siRNA experiments. Azidothymidine (AZT) and Efavirenz (EFA) were obtained from NIH AIDS Reagent Program (Germantown, MD, USA) and were dissolved in dimethyl sulfoxide (DMSO) (Sigma-Aldrich, St. Louis, MO, USA). 50 μg/ml AZT or EFA was used in infection experiments as controls. Inactivated virus control was made by heating virus at 65 °C for 1 h.

### Luciferase assay

Luciferase Assay System (Promega, Madison, WI, USA) was used and luciferase assay was performed according to the manufacturer’s instructions. Cells infected with HIV-1 Luc^+^ virus were washed with PBS, and then lysed with lysis buffer. After centrifugation at 15,000×g for 1 min, 20 μl of sample supernatant was mixed with 100 μl of Luciferase Assay Reagent. Luciferase activity was measured in Relative Light Units (RLU) by using a GloMax®-Multi Jr Single Tube Multimode Reader (Promega, Madison, WI, USA).

### Flow cytometry

Flow cytometry was used for both cell cycle analysis and quantification of infection. For cell cycle analysis by propidium iodide staining, cells were washed with PBS, fixed with ice-cold 70% ethanol, and stained with 0.1% (*v*/v) Triton X-100, 20 μg/ml propidium iodide (PI) (Sigma, St. Louis, MO, USA) and 100 μg/ml DNase-free RNase (Life Technologies, Grand Island, NY, USA). The Click-iT™ Plus EdU Flow Cytometry Assay Kit (Life Technologies, Grand Island, NY, USA) was also used to quantify S phase cells and the kit instruction was followed. For the infection assay, cells were disassociated by trypsin and washed with PBS. The infected GFP^+^ cells and uninfected cells were analyzed and quantified by a BD FACSVerse™ flow cytometer (BD Biosciences, San Jose, CA, USA). The FACSuite (BD Biosciences, San Jose, CA, USA) and the FlowJo (Ashland, OR, USA) software were used for data analysis.

### Real time PCR

For the quantification of late reverse transcription (RT) products, DNA was extracted from infected cells by using DNeasy Blood & Tissue Kit (Qiagen, Hilden, Germany, USA). The early, intermediate, late RT products and HIV-1 2-LTR cycle DNA were quantified by a TaqMan real time PCR, and the relative copy numbers were normalized to reference gene PBGD by using the ΔΔCt method [29]. The integrated HIV-1 provirus copy was also measured by a method described previously by our group. Sample DNA was amplified using the TaqMan Universal PCR Real time Reagent (Life Technologies, Grand Island, NY, USA) in a StepOne Plus real time PCR instrument (Life Technologies, Grand Island, NY, USA). StepOne software was used for quantitative analysis.

### Western blot

Proteins from cells were lysed with RIPA Lysis and Extraction Buffer (Thermos Fisher Scientific, Waltham, MA, USA). After being mixed with Laemmli buffer (BioRad, Hercules, CA, USA), protein samples were heated at 95 °C for 10 min. Protein samples were then separated by SDS-PAGE gel electrophoresis, and transferred onto PVDF membrane (Millipore, Billerica, MA, USA). After being probed with primary and secondary antibodies, protein bands in membranes were detected for chemiluminescence using SuperSignal™ West Pico Chemiluminescent Substrate (Thermo Fisher, Rockford, lL, USA). The primary antibodies used were: anti-p21^Cip1^ (#2947) and anti-phospho-SAMHD1 (Thr592) (#89930) (Cell Signaling Technologies, Danvers, MA, USA); anti-SAMHD1 (#12586–1-AP), anti-GAPDH (#60004–1-Ig) and anti-RRM2 (#11661–1-AP) (Proteintech Group, Inc. Rosemont, IL, USA); anti-p53 (#sc-126, Santa Crus, Dallas, TX, USA); anti-actin (#A5441, Sigma-Aldrich, St. Louis, MO, USA) and anti-tubulin (# N-356, Amersham, GE Healthcare, Pittsburgh, PA, USA). Western blot images were detected by the ChemiDoc XRS+ system (BioRad, Hercules, CA, USA), and image analysis was performed by using the Image Lab™ software (BioRad, Hercules, CA, USA).

### siRNA transfection

0.75 × 10^5^ HCT116 p53^+/+^ and HCT116 p53^−/−^ cells were cultured a 24 well plate overnight, siRNA transfections were performed using Lipofectamine® RNAiMAX™ Transfection Reagent (Life Technologies, Grand Island, NY, USA) according to the manufacturer’s instructions. After transfected with siRNA for 2 days, cells were cultured in DMEM without FBS for another 24 h before infection. siRNA transfection of hMDMs was performed two times with a recovery period of two days between transfections to ensure knockdown of target mRNA. siRNA knockdown was confirmed by Western blot. The Silencer Select validated siRNA siRNA p21^Cip1^ (#4390824) and negative control non-target siRNA (#4392420) were purchased from Life Technologies (Life Technologies, Grand Island, NY). siRNA p21 has a sequence of 5’-UAAAAUGUCUGACUCCUUGTT-3’. The FlexiTube siRNA p53 (# SI02655170) was purchased from Qiagen (Qiagen, Hilden, Germany, USA) and has a sequence of 5’- ACUCCACACGCAAAUUUCCTT-3’.

### Statistical tests

The Student’s t-test was used to evaluate the difference in copy numbers of RT in real time PCR, data from luciferase assay in infection quantification, and protein levels in Western blot experiments. *P*-values between 0.01 and 0.05, and less than 0.01 were considered significant and highly significant, respectively.

## Result

### The inhibition of HIV-1 infection in HCT116 p53^+/+^ was increased with cell cycle changed from cycling to non-cycling status

**Fig. 1:**
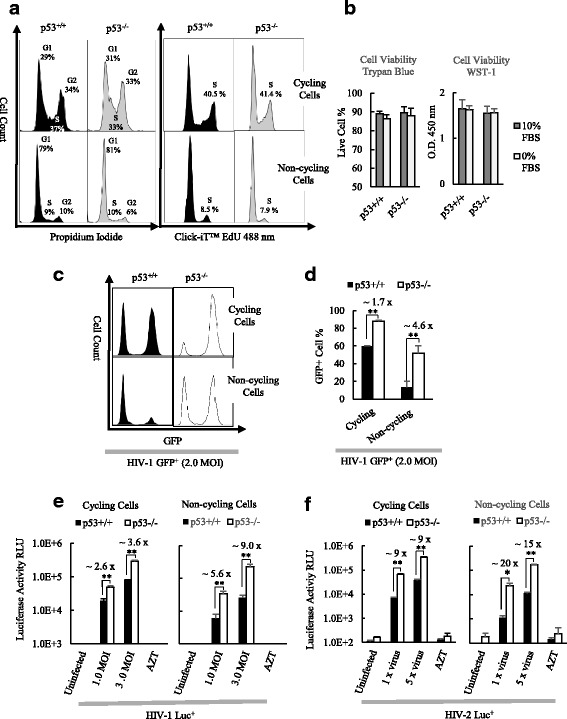
Comparison on the HIV infection between HCT116 p53^+/+^ and HCT116 p53^−/−^ cells in cycling and non-cycling status. **a** Flow cytometry analysis of cell cycle of cycling and non-cycling HCT116 p53^+/+^ and HCT116 p53^−/−^ cells. Non-cycling cells were cultured in DMEM without FBS for 24 h. Cells are stained with propidium iodide (PI) (Left). S phase cell percentages in cycling and non-cycling HCT116 p53^+/+^ and HCT116 p53^−/−^ cells were also analyzed by Click-iT™ Plus EdU Flow Cytometry Assay (Right). **b** Cell viabilities of both cycling and non-cycling HCT116 p53^+/+^ and HCT116 p53^−/−^ cells were measured by Trypan blue exclusion test (Left) and WST-1 assay (Right). **c** Flow cytometer analysis of infection 24 h after infection by VSV-G pseudotyped HIV-1 GFP^+^ virus in cycling and non-cycling HCT p53^+/+^ and HCT116 p53^−/−^ cells. Uninfected cells were shown as the 1st peak, and the infected GFP+ cells were shown as the 2nd peak. **d** Quantification of HIV-1 GFP^+^ infection in cycling and non-cycling HCT116 p53^+/+^ and HCT116 p53^−/−^ cells. **e** Quantification of infection of VSV-G pseudotyped HIV-1 Luc^+^ virus in cycling and non-cycling HCT116 p53^+/+^ and HCT116 p53^−/−^ cells. Luciferase assay for VSV-G pseudotyped HIV-1 Luc^+^ virus was performed at 24 h after infection. **f** Quantification of infection of VSV-G pseudotyped HIV-2 Luc^+^ virus in cycling and non-cycling HCT116 p53^+/+^ and HCT116 p53^−/−^ cells. 20 μl of transfection supernatant of pHIV-2 Luc^+^ and pVSV-G was used in 1 x virus infection; 100 μl of transfection supernatant was used in 5 x virus infection. Luciferase assay was performed at 48 h after HIV-2 Luc^+^ infection. The results represented a triplicate experiment. In Student’s t-test, *p* value < 0.05 is indicated by *; *p* value < 0.01 is indicated by **

It was found by our group that the infection of MLV vector based retrovirus was inhibited significantly in non-cycling HCT116 p53^+/+^, while the inhibition was attenuated in non-cycling HCT116 p53^−/−^ cells [26]. To investigate whether p53 also inhibits HIV-1 infection depending on cell cycle status, non-cycling HCT116 p53^+/+^ and HCT116 p53^−/−^ cells were prepared by serum starvation for 24 h, then the cell cycle statuses of both cycling and non-cycling cells were analyzed by flow cytometry after PI staining and EdU incorporation. The majority of HCT116 p53^+/+^ and HCT116 p53^−/−^ cells were in S and G2 phases when cultured in complete medium with 10% FBS (Fig. 1a). After being cultured in DMEM without FBS for 24 h both HCT116 p53^+/+^ cells and HCT116 p53^−/−^ cells became non-cycling cells, with very low percentages of cell populations in S phase and G2 phase (≤10%) (Fig. 1a). The cell viabilities of both cycling and non-cycling HCT116 p53^+/+^ and HCT116 p53^−/−^ cells were analyzed by trypan blue exclusion test and by WST-1 assay (Fig. 1b). The results showed that there were no cell viability differences between cycling and non-cycling cells in HCT116 p53^+/+^ and in HCT116 p53^−/−^ before infection.

Both cycling and non-cycling HCT116 p53^+/+^ and HCT116 p53^−/−^ cells were infected by 2.0 MOI VSV-G pseudotyped HIV-1 GFP^+^ virus (Fig. 1c and d). In cycling cell status both HCT116 p53^+/+^ and HCT116 p53^−/−^ were highly permeable to HIV-1 infection, and the infection in HCT116 p53^+/+^ cells were inhibited by about 1.7 fold in comparison to HCT116 p53^−/−^ cells. However the fold of inhibition in HCT116 p53^+/+^ increased to 4.6 times in non-cycling cells (Fig. 1c and d). To confirm this observation, both cycling and non-cycling HCT116 p53^+/+^ and HCT116 p53^−/−^ cells were infected by 1.0 and 3.0 MOI of VSV-G pseudotyped HIV-1 Luc^+^ virus respectively. In 1.0 MOI HIV-1 infection, the inhibition changed from about 2.6 fold to 5.6 fold, and in 3.0 MOI infection, the observed inhibition in HCT116 p53^+/+^ cells increased from 3.6 fold to 9 fold in comparison to HCT116 p53^−/−^ cells when cell cycle switched from cycling to non-cycling status (Fig. 1e). The amount of infection was dependent on virus dosage and no luciferase activities were detected in both uninfected cells and AZT treated cells. These results indicated the HIV-1 infection can be blocked by the presence of p53, and p53 dependent inhibition was increased with cell cycle change from cycling to non-cycling status.

In order to investigate whether HIV-2 infection is also inhibited by p53 dependent on cell cycle status, VSV-G pseudotyped HIV-2 Luc^+^ virus was used to infect both cycling and non-cycling HCT116 p53^+/+^ and HCT116 p53^−/−^ cells (Fig. 1f). HIV-2 infection was significantly inhibited in HCT116 p53^+/+^ in comparison to HCT116 p53^−/−^ cells. The inhibition in HCT116 p53^+/+^ was about 9 folds in cycling cells when infected with 1 x and 5 x HIV-2 Luc^+^ viruses respectively, and inhibition in HCT116 p53^+/+^ was about 20 and 15 folds in non-cycling cells when infected with 1 x and 5 x HIV-2 Luc^+^ viruses respectively (Fig. 1f). This finding suggested the inhibition to overall HIV-2 infection was increased compared to the inhibition to HIV-1 infection in HCT116 p53^+/+^, and the inhibition to HIV-2 was also increased with cell cycle change from cycle to non-cycling status.

### HIV-1 reverse transcription was inhibited in HCT116 p53^+/+^ cells compared to HCT116 p53^−/−^ cells in non-cycling cell cycle status

**Fig. 2:**
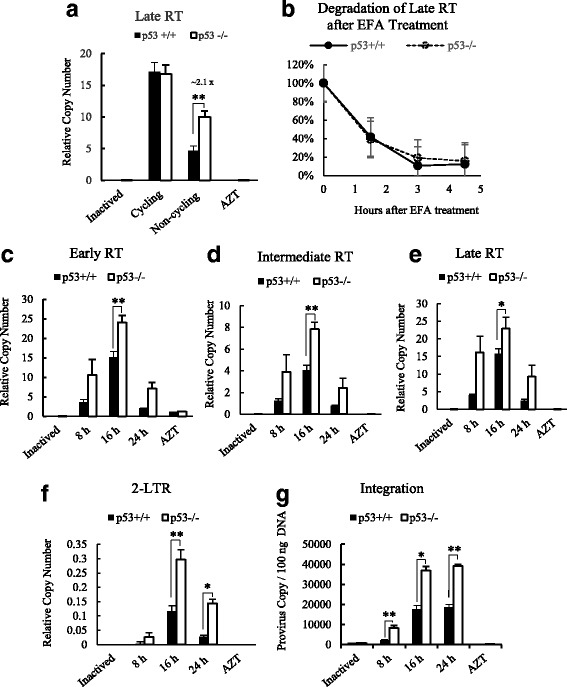
Comparison of HIV-1 reverse transcription and viral cDNA degradation in infected HCT116 p53^+/+^ and HCT116 p53^−/−^ cells. **a** Quantification of HIV-1 late RT in infected cycling HCT116 p53^+/+^ and HCT116 p53^−/−^ cells and non-cycling HCT116 p53^+/+^ and HCT116 p53^−/−^ cells 16 h post infection. **b** Evaluation of HIV cDNA degradation after treatment of the reverse transcriptase inhibitor EFA. The relative copy number of HIV-1 late RT product (viral cDNA) was quantified by TaqMan real time PCR. At each time point, the remaining viral cDNA (%) was calculated from the copy of cDNA per cell treated with EFA divided by the copy of cDNA per untreated cell. Time indicates the number of hours after the addition of EFA. The results represented a triplicate experiment. Comparison of HIV-1 cDNA at different stages of virus replication between non-cycling HCT116 p53^+/+^ and non-HCT116 p53^−/−^ cells at 8 h, 16 h and 24 h post infection were performed. **c** Early RT. **d** Intermediate RT. **e** Late RT. **f** 2-LTR cycle DNA. **g** Integration. Both inactived virus and AZT treatment were used as negative control. Relative RT copy numbers were quantified by a TaqMan real time PCR. In Student’s t-test, *p* value < 0.05 is indicated by *; *p* value < 0.01 is indicated by **

We found previously that p53 inhibited retrovirus reverse transcription [26]. To identify whether p53 dependent HIV-1 inhibition in non-cycling cells also happened at reverse transcription, a TaqMan real time PCR was used to quantify HIV-1 reverse transcription (RT) late product in infected cells. It was found there was no difference in amount of RT products between cycling HCT116 p53^+/+^ and cycling HCT116 p53^−/−^ cells at 16 h post infection, however HIV-1 RT product was decreased about 2.1 times in HCT116 p53^+/+^ cells compared to HCT116 p53^−/−^ cells in non-cycling cells (Fig. 2a). In order to identify whether the decrease of RT product in HCT116 p53^+/+^ cells was either due to the inhibition in the process in viral cDNA synthesis by reverse transcriptase or because of possible degradation of virus cDNA by nucleases in infected cells, the amount of remaining late RT product was quantified at 1.5 h, 3.0 h and 4.5 h after cells were treated with the reverse transcriptase inhibitor EFA at 12 h post infection. No difference was observed in the percentages of virus cDNA degradation between non-cycling HCT116 p53^+/+^ and HCT116 p53^−/−^ cells after blocking reverse transcriptase by EFA (Fig. 2b). This result indicated that the observed p53 dependent inhibition occurred in the synthesis of virus cDNA by HIV-1 reverse transcriptase. In a separate HIV-1 infection experiment, the amount of virus cDNA at different reverse transcription stages were quantified (Fig. 2c-e). HIV-1 early, intermediate and late reverse transcription products were found to be consistently decreased in non-cycling HCT116 p53^+/+^ cells compared to non-cycling HCT116 p53^−/−^ cells over 8 h, 16 h and 24 h after infection (Fig. 2c-e). HIV-1 2-LTR cycle DNA and integration were also quantified (Fig. 2f and g). The amount of decrease of 2-LTR cycle DNA and integrated HIV-1 provirus copy in non-cycling HCT116 p53^+/+^ cells in comparison to non-cycling HCT116 p53^−/−^ cells were about 2 times less, which are similar to the observed level of inhibitions in different RT stages. These results indicated that the block of HIV-1 infection in non-cycling HCT116 p53^+/+^ cells occurred at the reverse transcription stage mostly by a mechanism involving in the processing of HIV-1 reverse transcription.

### The p53 and its downstream gene p21 proteins were increased in HCT116 p53^+/+^ when cell cycle was changed from cycling to non-cycling status

**Fig. 3:**
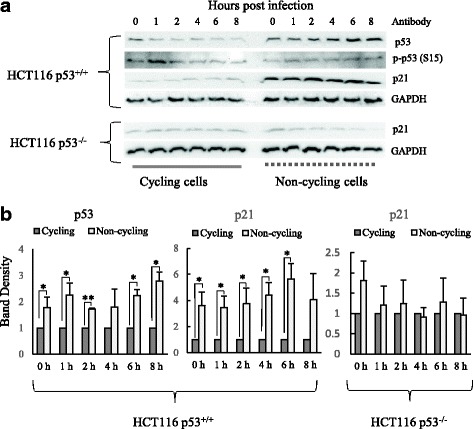
Western Blot Analysis of p53, p-p53 (S15) and p21 proteins in HIV-1 infected HCT116 p53^+/+^ and HCT116 p53^−/−^ cells when cells changes from cycling cells to non-cycling cells. **a** Western blot of p53, p-p53 (S15) and p21 in HIV-1 infected cycling and non-cycling HCT116 p53^+/+^ cells and HCT116 p53^−/−^ cells. **b** Quantification of Western blot of p53, p-p53 (S15) and p21 proteins. The results represented a duplicate experiment. In Student’s t-test, *p* value < 0.05 is indicated by *

To investigate the p53 gene function change when cell cycle was changed from cycling to non-cycling status, and its response to HIV-1 infection, Western blot experiments were carried out to analyze the changes of p53, phosphorylated p-p53(S15) and the p53 downstream gene p21 (Fig. 3a and b). It was found that both the levels of p53 protein and p21 protein were significantly increased in non-cycling HCT116 p53^+/+^ cells in comparison to cycling HCT116 p53^+/+^ cells (Fig. 3a and b). p-p53 (S15) was slightly elevated at 1 h after HIV-1 infection in cycling HCT116 p53^+/+^ cells, but no difference of p-p53 (S15) was found in non-cycling HCT116 p53^+/+^ cells. Western blots were also performed on cycling and non-cycling HCT116 p53^−/−^ cells in the course of HIV-1 infection (Fig. 3a and b). The expression level of p21 was low in HCT116 p53^−/−^ cells and there were no significantly difference in p21 protein levels in HCT116 p53^−/−^ cells between cycling and non-cycling cells. This data suggests that the cell cycle status change resulting from serum starvation induced p53 expression, which in turn regulated the expression of p21 in HCT116 p53^+/+^ cells. However the increase of p21 resulting from cell cycle change did not occur in HCT116 p53^−/−^ cells, which is in concordance with the lack of regulation by a functional p53.

### The analysis of restriction to HIV-1 infection by p53 and p21 in non-cycling HCT116 p53^+/+^ cells

**Fig. 4:**
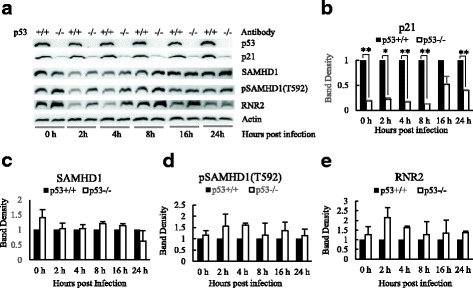
Comparative Analysis of p21, SAMHD1, pSAMHD1 (T592) and RNR2 between non-cycling HCT116 p53^+/+^ cells and non-cycling HCT116 p53^−/−^ cells in HIV-1 infection. **a** Western blot of p21, SAMHD1, pSAMHD1 (T592) and RNR2 between non-cycling HCT116 p53^+/+^ cells and non-cycling HCT116 p53^−/−^ cells in HIV-1 infection. **b** Quantitative analysis of p21 protein between non-cycling HCT116 p53^+/+^ cells and non-cycling HCT116 p53^−/−^ cells in HIV-1 infection. **c** Quantitative analysis of SAMHD1 protein between non-cycling HCT116 p53^+/+^ cells and non-cycling HCT116 p53^−/−^ cells in HIV-1 infection. **d** Quantitative analysis of pSAMHD1 (T592) protein between non-cycling HCT116 p53^+/+^ cells and non-cycling HCT116 p53^−/−^ cells in HIV-1 infection. **e** Quantitative analysis of RNR2 protein between non-cycling HCT116 p53^+/+^ cells and non-cycling HCT116 p53^−/−^ cells in HIV-1 infection. The results represented a duplicate experiment. In Student’s t-test, *p* value < 0.05 is indicated by *; *p* value < 0.01 is indicated by **

It has been shown that p21 inhibits HIV reverse transcription by regulating cellular dNTPs level in differentiated macrophages. p21 was found to phosphorylate SAMHD1 at Thr592 [20], which inactivates its activity to degrade cellular dNTPs. p21 was also reported to down regulate expression of RNR2 [17], an enzyme responsible for cellular synthesis of dNTPs. Western blot experiments were carried out to compare p21, SAMHD1, pSAMDH1(T592) and RNR2 protein levels between non-cycling HCT116 p53^+/+^ and non-cycling HCT116 p53^−/−^ cells (Fig. 4a). The levels of p21 protein were significantly higher over the course of HIV-1 infection in non-cycling HCT116 p53^+/+^ in comparison to non-cycling HCT116 p53^−/−^ cells (Fig. 4a and b). There was no difference found in overall SAMHD1 levels (Fig. 4a and c), however both pSAMDH1 (T592) and RNR2 protein levels were elevated over multiple time points over HIV-1 infection in non-cycling HCT116 p53^+/+^ in comparison to non-cycling HCT116 p53^−/−^ cells (Fig. 4a and d, e), even though the differences were not statistically significant.

### siRNA knockdown of either p53 or p21 rescued HIV-1 reverse transcription from the inhibition in non-cycling HCT116 p53^+/+^ cells

**Fig. 5:**
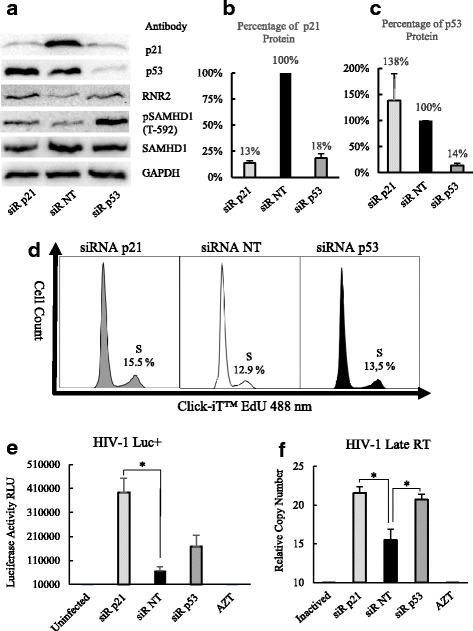
The analysis of HIV-1 infection after siRNA knockdown of p53 and p21 in non-cycling HCT116 p53^+/+^ cells. **a** Western blot of p53, p21, RNR2, SAMHD1 and pSAMHD1(T-592) proteins in non-cycling HCT116 p53^+/+^ cells after siRNA transfection. **b** Quantification of percentage of p21 after siRNA knockdown of p21 and siRNA knockdown of p53. **c** Quantification of percentage of p53 after siRNA knockdown of p21 and siRNA knockdown of p53. **d** Cell cycle analysis after siRNA transfections by Click-iT™ Plus EdU Flow Cytometry Assay. **e** Quantification of infection in non-cycling HCT116 p53^+/+^ cells after siRNA knockdown of p21 and siRNA knockdown of p53. **f** Quantification HIV-1 RT in non-cycling HCT116 p53^+/+^ cells after siRNA knockdown of p21 and siRNA knockdown of p53. 2.0 MOI HIV-1 was used to infect non-cycling HCT116 p53^+/+^ cells in the quantification of infection and late RT. The non-targeting (NT) siRNA was used as negative control. HIV-1 RT was quantified by TaqMan real time PCR at 16 h post infection. The results represented a triplicate experiment. In Student’s t-test, *p* value < 0.05 is indicated by *

To further verify that the increase in p53 and p21 at protein level was responsible for the inhibition of HIV-1 reverse transcription in non-cycling cells, HCT116 p53^+/+^ cells were transfected with either p53 siRNA or p21 siRNA. The non-targeting (NT) siRNA was used as negative control. After 48 h the medium were replaced with DMEM without FBS and cultured for another 24 h, and then siRNA transfected cells were infected with HIV-1 virus. As shown in Fig. 5a, both knockdown of p53 and knockdown of p21 by siRNA transfection were confirmed by Western blot experiment (Fig. 5a). Transfection with p21 siRNA resulted in knockdown of p21 to 13% (Fig. 5b). Transfection with p53 siRNA also led to knockdown of p21 to 18%. Transfection with p53 siRNA resulted in knockdown of p53 to 14% (Fig. 5c). To analyze the potential role of p53 and p21 in the regulation of genes responsible for maintaining host cell dNTPs pool size, SAMHD1, pSAMHD1(T592) and RNR2 protein levels were compared between HCT116 p53^+/+^ cells transfected with p53 or p21 gene specific siRNA and non-targeting siRNA (Fig. 5a). It was found that the knockdown of p53 increased both RNR2 and pSAMHD1 (T-592), and the knockdown of p21 increased RNR2 and increased pSAMHD1 (T-592) slightly as well. Either the knockdown of p53 or the knockdown of p21 did not change SAMHD1 at protein level significantly (Fig. 5a). The cell cycle status after siRNA treatment was also tested by Click-iT™ Plus EdU Flow Cytometry Assay. There were a minor increase in S phase in siRNA p21 treated cells (15.5%) and siRNA p53 treated cells (13.5%) compared to siRNA NT treatment (12.9) (Fig. 5d). siRNA knockdown of p21 in non-cycling HCT116 p53^+/+^ cells significantly increased infection of HIV-1 Luc^+^ virus, and siRNA knockdown of p53 in non-cycling HCT116 p53^+/+^ cells also increased infection of HIV-1 Luc^+^ virus but the increase was not statistically significant (Fig. 5e). Meanwhile, either p53 siRNA or p21 siRNA treatment significantly increased HIV-1 reverse transcription in non-cycling HCT116 p53^+/+^ cells (Fig. 5f). The above data indicated that it was the elevated p53 protein or p21 protein in non-cycling HCT116 p53^+/+^ cells that caused the inhibition in HIV-1 reverse transcription.

### The inhibition of HIV infection by p53 and its downstream gene p21 in hMDMs

**Fig. 6:**
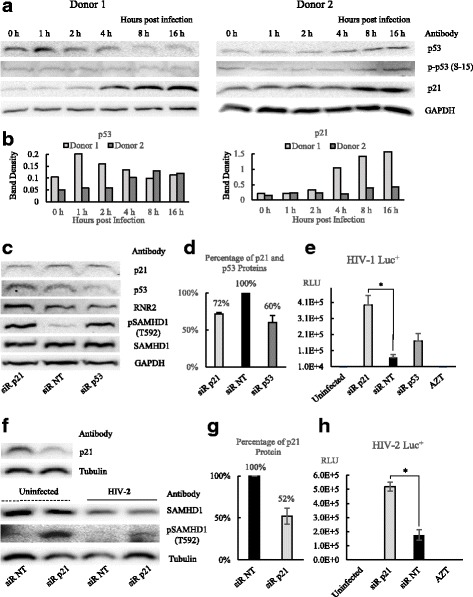
Analysis of function of p53 and its downstream gene p21 during HIV infection in hMDMs. **a** and **b** Analysis of p53, p-p53 (S15) and p21 proteins in hMDMs after HIV-1 infection by Western blot. 3.0 MOI VSV-G pseudotyped HIV-1 GFP^+^ virus was used to infect 0.5 × 10^6^ hMDMs in a 24 well plate, Results from two different donors were shown. **c** Western blot analysis of SAMHD1, pSAMHD1 (T592) and RNR2 after siRNA knockdown of p53 and siRNA knockdown of p21 in hMDMs. **d** Percentages of p21 protein and p53 protein after siRNA knockdown. The non-targeting (NT) siRNA was used as negative control. **e** Quantification of HIV-1 Luc^+^ infection after siRNA knockdown of p21 and siRNA knockdown of p53 in hMDMs. **f** Analysis of SAMHD1 and pSAMHD1(T592) in HIV-2 infection after siRNA knockdown of p21 in hMDMs. **g** The percentage of p21 after siRNA knockdown in hMDMs. **h** Quantification of HIV-2 infection in hMDMs transfected with p21 siRNA and non-targeting siRNA. The non-targeting (NT) siRNA was used as negative control. Luciferase assay was performed at 48 h after HIV-2 Luc^+^ infection. The results represented a triplicate experiment. In Student’s t-test, *p* value < 0.05 is indicated by *

To identify whether p53 and p21 impact HIV-1 infection in its natural host cells, VSV-G pseudotyped HIV-1 Luc^+^ was used to infect hMDMs. Protein levels of p53, p-p53(S-15) and p21 were analyzed by Western blot at multiple time points during the early stage of HIV-1 replication (Fig. 6a and b). In donor 1, p53 protein level was increased at 1 h after infection. The amount of p21 protein started to increase at 2 h post infection, and higher amounts of p21 proteins were found as early as 4 h and 8 h post infection. In donor 2, p21 protein was also increased at time points of 8 h and 16 h after infection, and there was minor increase in p53 proteins at time points of 4 h, 8 h and 16 h. No noticeable changes were observed in p-p53 (S-15) in infected hMDMs. Interestingly, p21 protein levels were increased in hMDMs in both donors at an early stage of HIV-1 replication, approximately when reverse transcription is in process. This result strongly suggests the role of p21 in the restriction to early stage of HIV-1 replication in hMDMs.

The function of p53 and p21 in HIV-1 infection was further examined by siRNA experiments. After the siRNA transfection, p21 protein level dropped about 28% in cultured hMDMs (Fig. 6c and d). The knockdown of p21 increased both RNR2 and pSAMHD1(T592), and HIV-1 infection increased significantly (Fig. 6c and e). After siRNA transfection, p53 protein level dropped about 40% in cultured hMDMs. The knockdown of p53 increased pSAMHD1(T592), and HIV-1 infection was increased but not statistically significant (Fig. 6c-e). There were no obvious changes in RNR2 protein levels after knockdown of p53, and knockdown of either p21 or p52 did not alter total SAMHD1 protein levels.

p21 was previously found to restrict HIV-2 infection [15, 16]. The Vpx protein from HIV-2 is able to degrade cellular restriction factor SAMHD1 [30], while p21 can inactivate SAMHD1 by phosphorylation [20]. To investigate the role of p21 in HIV-2 infection, hMDMs were transfected with p21 siRNA, then infected by HIV-2 Luc^+^ virus. In this experiment siRNA transfection was able to knockdown p21 to about 52% (Fig. 6f, g). In control hMDMs transfected with non-targeting siRNA, HIV-2 infection resulted in the decrease in SAMHD, and the levels of pSAMHD1 (T592) were almost undetectable before and after HIV-2 infection (Fig. 6f). p21 siRNA knockdown did not change SAMHD1 levels but greatly increased pSAMHD1 (T592) in both HIV-2 infected and uninfected hMDMs (Fig. 6f). Finally it was found that knockdown p21 in hMDMs significantly increased HIV-2 infection (Fig. 6h). This result also suggested that the degradation of SAMHD by HIV-2 Vpx was independent from phosphorylation of SAMHD1 resulted from p21 knockdown by siRNA in hMDMs.

## Discussion

The permissiveness of HIV infection is dependent on the host cell’s cell cycle status. Activated macrophages and proliferating CD4^+^ T lymphocytes are highly susceptible to infection, however resting CD4^+^ T cells and quiescent macrophages are largely non-permissive to HIV-1 replication [31–33]. Early studies demonstrated that restriction to HIV-1 infection in non-cycling quiescent macrophages and resting CD4^+^ T cells occurred during reverse transcription [33–35]. Korin found HIV infection was successful only in CD4^+^ T cells that transited into the G1b phase of the cell cycle [36]. Mlcochova et al.... reported recently that HIV-1 infection was highly susceptible in stimulated G1-like phase macrophages, which are characterized by an increase in D-type cyclins, upregulation of CDK1 with subsequent SAMHD1 T592 phosphorylation [37]. Histone deacetylase inhibitors (HDACi) treatment blocked the transition from G1-like phase to a non-permissive state. The block by HDACi in hMDMs was associated with increased expression of p53 [37]. p21 is a well-known CDK inhibitor, and it functions to block cell cycle transition at G1 when being activated [38]. Our results suggest that the response of p53 and its downstream gene p21 to cell cycle status changes will significantly impacted the host cell’s permissiveness to HIV-1 infection.

We found that HIV-1 infection was inhibited in HCT p53^+/+^ cells in comparison to HCT p53^−/−^ cells. In cycling cells inhibitions to HIV-1 infection were 1.7 fold (2.0 MOI HIV-1 GFP^+^), 2.6 fold (1.0 MOI HIV-1 Luc+) and 3.6 fold (3 MOI HIV-1 Luc+) respectively, while in non-cycling cells inhibitions were more than doubled, i.e. 4.6 fold (2.0 MOI HIV-1 GFP^+^), 5.6 fold (1.0 MOI HIV-1 Luc+) and 9.0 fold (3.0 MOI HIV-1 Luc+) respectively. It has been known that p53 inhibits HIV-1 infection at transcription level [3, 5, 6], which is the inhibition to the late stage of HIV replication and will applies to HIV-1 infections in both cycling and non-cycling cells. Our findings pointed out that the increased inhibition in HCT p53^+/+^ non-cycling cells was due to the additional block in the reverse transcription.

p53 expression was elevated when the cell cycle switched from cycling to non-cycling after serum starvation (Fig. 3), which agrees with the previous findings by both Shang et al... and Shi et al that serum starvation can induce p53 expression [39, 40]. The increased p53 expression subsequently induced the expression of its downstream gene p21. The siRNA knockdown experiment confirmed that p53 and p21 were responsible for the observed inhibition in HIV-1 reverse transcription (Fig. 4). We also found for the first time that p53 and p21 increased at protein level in hMDMs at very early time (from 1 to 8 h after infection) during HIV-1 infection. These data strongly indicated that p53 and its downstream gene p21 play an important role in the restriction of HIV-1 early stage replication in natural host cells.

In this study we also investigated the host cell’s restriction to HIV infection by regulating the level of cellular dNTPs. SAMHD1 is a cellular dNTPase that restricts HIV infection by lowering cellular dNTPs to a level required for reverse transcription. RNR2 is responsible for the de novo synthesis of dNTPs. The two main enzymes controlling dNTP pool sizes are adjusted to the requirements of DNA replication following cell cycle in mammalian cells [41]. Allouch et al.... reported after p21 was induced by immune complex aggregation of FcγRs in macrophages, it restricted HIV reverse transcription by blocking the synthesis of dNTPs through the inhibition of the expression of RNR2 [17, 21]. Pauls et al found that in macrophages differentiated by M-CSF p21 blocked the phosphorylation of SAMHD1 [20]. Phosphorylation inactivates the dNTPase activity of SAMHD1 [20]. We found that the knockout of p53, and siRNA knockdown of p53 and p21 were associated with the increase protein levels of both RNR2 and pSAMHD1 (T592) in non-cycling HCT116 p53^+/+^ cells (Figs. 4 and 5). Furthermore, the siRNA knockdown of p21 in hMDMs increased protein levels of both RNR2 and pSAMHD1 (T592) (Fig. 6c). Our data highly suggested that p21 could suppress the expression of RNR2 and block the phosphorylate SAMHD1 simultaneously in non-cycling cells so as to restrict HIV-1 infection through regulating cellular dNTPs. We also found that siRNA knockdown of p53 induced pSAMHD1 (T592) in both HCT116 p53^+/+^ cells and hMDMs. (Figs. 5 and 6c). Micochova reported recently that HDACi can block HIV-1 infection by inhibiting phosphorylation of SAMHD1 via p53 activation [37]. It remains to be elucidated whether p53 can inhibit the phosphorylation of SAMHD1 independent of p21.

p21 may also inhibit HIV infection by pathways other than through regulating the cellular level of dNTPs. p21 was found to inhibit reverse transcription by phosphorylating HIV-1 reverse transcriptase [18]. Others showed that the restriction to HIV infection by p21 was associated with Vpr [42–44]. We found that p53 and p21 inhibited HIV-2 infection in both cycling and non-cycling HCT116 p53^+/+^ cells, and the siRNA knockdown of p21 increased HIV-2 infection in hMDMs. These results agrees with the findings by Bergamaschi et al and Allouch et al that p21 restricted HIV-2 and SIV infection [16, 17]. Since Vpx proteins in HIV-2 and SIV are able to target SAMHD1 for proteasomal degradation [30, 45, 46], the p21 dependent block of HIV-2 in hMDMs suggested a mechanism of host restriction that is less dependent on the level of cellular dNTPs.

## Conclusions

HIV infection in macrophages is highly relevant to AIDS pathogenesis. Human macrophages have been described as a durable reservoir of HIV-1 characterized by the persistence of viral replication and the poor susceptibility to antiviral therapy [47, 48]. The transmission of HIV-1 from macrophages to CD4+ T lymphocytes can be achieved by efficient cell-to-cell transmission even when there is inadequate amount of cell-free virus in environment [48]. Here we show for the first time that p53 and its downstream gene p21 were induced in hMDMs in the early stage of HIV infection, and the elevated p53 and p21 inhibited reverse transcription. The underlined mechanism obtained in this study will shed light on the understanding of HIV pathogenesis, and control and management of AIDS disease.

## Abbreviations

FBS: Fetal bovine serum
HIV-1: The human immunodeficiency virus type 1
HIV-2: The human immunodeficiency virus type 2
hMDMs: Human monocyte derived macrophages
LTR: long terminal repeat sequence
p21: the cyclin-dependent kinase inhibitor p21^Waf1/Cip1^
p53: Tumor protein p53
PI: Propidium iodide
PKR: Double-stranded RNA (dsRNA)-dependent protein kinase
RNR2: Ribonucleotide reductase subunit R2
RT: HIV-1 reverse transcription
RT: Reverse transcription
SAMHD1: SAM domain and HD domain-containing protein 1
VSV-G: Vesicular stomatitis virus G-protein
